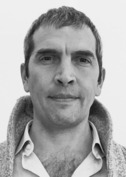# From Chemical Mutagenesis to Post‐Expression Mutagenesis: A 50 Year Odyssey

**DOI:** 10.1002/anie.201509310

**Published:** 2016-04-27

**Authors:** Tom H. Wright, M. Robert J. Vallée, Benjamin G. Davis

**Affiliations:** ^1^ Department of ChemistryUniversity of OxfordChemistry Research LaboratoryMansfield RoadOX1 3TAUK

**Keywords:** amino acids, mutagenesis, peptides, protein modifications, synthetic biology

## Abstract

Site‐directed (gene) mutagenesis has been the most useful method available for the conversion of one amino acid residue of a given protein into another. Until relatively recently, this strategy was limited to the twenty standard amino acids. The ongoing maturation of stop codon suppression and related technologies for unnatural amino acid incorporation has greatly expanded access to nonstandard amino acids by expanding the scope of the translational apparatus. However, the necessity for translation of genetic changes restricts the diversity of residues that may be incorporated. Herein we highlight an alternative approach, termed post‐expression mutagenesis, which operates at the level of the very functional biomolecules themselves. Using the lens of retrosynthesis, we highlight prospects for new strategies in protein modification, alteration, and construction which will enable protein science to move beyond the constraints of the “translational filter” and lead to a true synthetic biology.

## 1 Introduction

The detailed, mechanistic interrogation of protein function with residue‐level precision requires methods for the conversion of one amino‐acid side chain into another (one residue into another) and the assembly of proteins which contain such precise changes. To date, site‐directed gene mutagenesis has provided the primary technology for achieving this goal.^1^ Gene‐level mutagenesis is founded on the central dogma of Francis Crick, which describes the directional flow of information in biological macromolecules from DNA to protein.^2^ By using this form of site‐directed mutagenesis, information is modified at the nucleotide level to ultimately effect a change in protein sequence, and hence at the functional biomolecule level. Through the suppression of certain codons^3^ with tRNAs loaded with unnatural amino acids, unnatural amino acids can be introduced at a specific position of a protein, for example, by stop codon positioning^4^ if a specific, paired tRNA‐tRNA synthetase system can be engineered.^5^ This approach is an exciting and strikingly powerful method for certain structural motifs which are well‐tolerated by the corresponding biosynthetic systems (e.g., synthetases, ribosome). However, one shortcoming of such (stop)codon suppression methodology is the lack of chemical diversity in the residues beyond those that can be incorporated with current technology, largely reflecting the evolved stringency of the amino‐acid *t*RNA synthetase proteins. Most amino acids that have been incorporated thus far resemble lysine or tyrosine (by stop‐codon) or methionine (by Met‐codon) derivatives, thus reflecting the natural propensities of the most common synthetases that have been exploited.^3^ Notably, there have been no synthetases reported for the direct incorporation of certain residues that are central to much current biological exploration. Such residues are the methylated derivatives of lysine, glutamine, and arginine, which are widely implicated in epigenetics or for any glycosylated amino acids. Indeed, whilst there have been some elegant indirect strategies adopted,^6^ the difficulty of engineering a synthetase that can either discriminate a single methyl group and solely charge the modified residue, or accommodate even a single sugar on a side chain (let alone the larger complex structures found in glycobiology) is daunting. Despite these shortcomings, the success of these technologies is illustrated by their daily use in countless labs around the world.

In some respects, approaches such as these, which are transmitted (transcripted and translated=expressed) from the nucleotide level, are a fundamentally circuitous means of achieving what is really sought after—a change in a specific side chain at the protein level. In principle, a method for post‐translational or post‐expression mutagenesis would be able to accomplish this transformation directly, at the level of the protein itself. Such a method would not be constrained by the evolutionary “filter” of the translational apparatus and would enable site‐selective installation of any desired side chain, natural or designed, straight into the biomolecules that actually directly determine function in biology (the proteins and even the sugars and lipids).

**Scheme 1:**
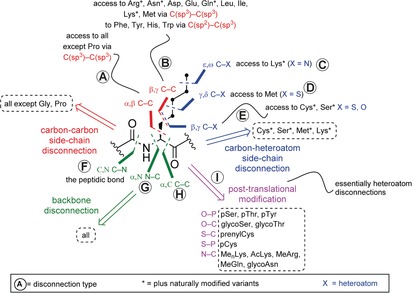
Retrosynthetic analysis of protein construction.

In nature too, post‐translational modification (PTM) is the key means by which protein side‐chain diversity is extended beyond the proteinogenic amino acids. Chemically simple modifications such as methylation, and more complex structures, such as the attachment of sugars to glycoproteins, have profound consequences for biological processes in all kingdoms of life. The biochemical study of PTMs has been hampered by the lack of general and robust methods for their selective installation to a protein. The post‐translationally modified side chains pose a distinct challenge for gene‐level mutagenesis. An alternative approach to an expanded mutagenesis could draw synthetic inspiration from this natural strategy of PTM (Scheme 1, disconnection I) and modify proteins directly, without genetic mutation, in a test tube and ultimately inside cells and organisms.

A number of biochemists, in the mid‐to‐late 1960s, who were working quietly before the biotechnological revolution of molecular biology and amongst the more vocal backdrop of peptide synthesis, envisaged a synthetic approach to mutagenesis as a valuable tool for probing protein function. This work, which led to the first far‐sighted conversion of one amino acid into another through the chemical alteration of a residue side chain (from Ser‐to‐Cys),^7^ demonstrated a key principle. In this way, a powerful conceptual legacy for a more general method that could be applied directly to biomolecules was established. Indeed, this approach enabled the first point (site‐directed protein) mutations of an enzyme, by any method, by using just such a synthetic, chemical approach. Polgar and Bender used the term “simulated mutations” and described a hypothetical method: “……by chemical or enzymatic means it is possible to substitute one amino acid of the protein molecule by another, simulating the effect of mutation.”7b

## 2 The Early Years: β,γ‐Carbon–Heteroatom Bond Formation

**Scheme 2:**
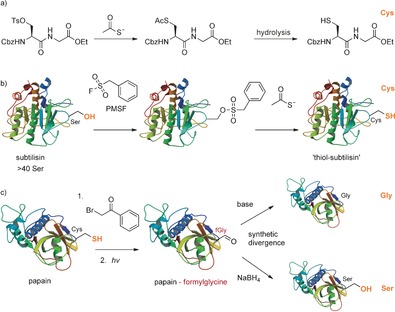
Pioneering examples of β,γ‐carbon–heteroatom bond formation.

**Scheme 3:**
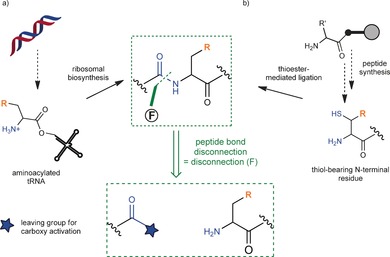
Peptide bond disconnection for inserting an amino acid of choice. As the dominant mode of protein synthesis (chemically and biosynthetically), strategic methods for creating sources of fragments are widespread. Here are two contrasting methods for generating an amino acid of choice. Both exploit the nucleophilic C‐terminal synthetic equivalent fragment which is revealed by disconnection F: a) A “charged” tRNA used during N‐to‐C ribosomal biosynthesis (and so must be tolerated by the ribosome). b) A C‐terminal peptide fragment that bears a thiol auxiliary which may participate in thioester‐mediated NCL with a suitably activated N‐terminal carboxy donor fragment.

We can trace the first roots of the chemical mutagenesis concept to a 1965 paper from Wilchek et al., in which serine residues were quantitatively converted into cysteines in polypeptides (Scheme 2 a).7a Serine tosylation and subsequent S_N_2 displacement enabled the synthesis of both cysteine (reaction with thioacetate followed by hydrolysis) and unnatural analogues (reaction with other thiol nucleophiles), thus demonstrating the advantages of the chemical approach for rapid diversification of side‐chain structures. The powerful strategic leap embodied in this work can be analyzed through simple retrosynthetic analysis of a generic polypeptide structure. It was the case then (and remains the case now, some would argue) that the dominant disconnection of protein synthesis has been that of the peptidic bond (Scheme 1, disconnection F and see Scheme 3). This disconnection reflects not only nature's primary synthetic method (amino‐acid ester + peptide amine=amide) but also chemical methods through other activated carboxy derivatives of amino acids.^8^ Wilchek et al. (Scheme 1, disconnection E) ushered in the notion that other disconnections would and should prove powerful, and would offer additional synthetic flexibility in the creation of proteins.7a

Building on this prescient work, in 1966, the groups of Koshland and Bender independently reported the chemical transformation of one specific amino‐acid side chain into another on an intact protein, that is, the first point mutation, by any method (Scheme 2 b).7b,7c In both reports, the active‐site serine of subtilisin was chemically converted into a cysteine residue. By comparison, the first mutation of a protein achieved by DNA‐level site‐directed mutagenesis was not to be achieved until 1978.^9^ Sulfonylation of the active‐site serine with phenylmethanesulfonyl fluoride, to create a useful leaving group, enabled displacement by thioacetate and subsequent acetyl hydrolysis, likely through the inherent activity of the enzyme. This reaction allowed the creation of a thiol‐subtilisin, bearing a cysteine residue as the active‐site nucleophile instead of Ser. The presence of the desired cysteine was confirmed by amino‐acid analysis and colorimetric titration.

One potentially attractive factor missing in these early reports was the element of synthetic divergence from a suitable precursor. The work of Clark and Lowe^10^ on chemical mutations of papain provided the first example of mutational divergence in amino‐acid side‐chain conversion through the creation of both Gly and Ser from a common Cys precursor (Scheme 2 c). The nucleophilic cysteine of papain was chemically mutated via the intermediate formylglycine. Notably, formylglycine has itself since been recognized as a desirable residue, both as a natural PTM (derived enzymatically from Cys or Ser) mediating sulfate ester hydrolysis at the active site of type I sulfatases, and as a uniquely reactive aldehyde tag for further modification.^11^ Synthetically, chemical mutation of papain by Clark and Lowe commenced with selective alkylation of the reactive active‐site Cys using phenacyl bromide. Repeated photolysis was necessary to convert the phenacyl‐inhibited protein into the desired thioaldehyde product. The thioaldehyde slowly hydrolyzed, thus forming formylglycine with the release of hydrogen sulfide. From formylglycine, reduction with NaBH_4_ provided Ser while incubation at pH 9.0 for prolonged periods led to Gly by a retroaldol reaction.^10^

Whilst undeniably prescient work, it should be noted that the chemistry performed by the groups of Koshland, Bender, and Lowe exploited the enhanced nucleophilicity of the active‐site nucleophiles in subtilisin (Ser) and papain (Cys). This approach provides a salient illustration of two attributes which can together create synthetic utility: both chemo‐ and regioselectivity. Although, therefore, a powerful strategy for achieving regioselectivity, its necessarily limited scope (to a single active‐site residue in certain enzymes) does not provide a general solution with free‐ranging control of site selectivity.^12^

## 3 Retrosynthetic Analysis of Protein Modification

To overcome the limitations in generality of early strategies for chemical approaches to mutagenesis (lack of generality in the side chains which can be accessed and also sometimes harsh reaction conditions), the application of retrosynthetic logic, as a convenient tool for allowing one to escape occasional subjective narrowness of design,^13^ helps to identify further disconnections and thence synthons. For example, through the analysis of the early work on chemical mutagenesis described in the section above, we see that these E‐type disconnections (Scheme 1) deliver, in both cases, electrophilic protein synthons at Cβ matched with suitable Xγ nucleophiles. The candidate precursors that are the synthetic equivalents of such synthons are typically called tags. We have used the terminology of tag‐and‐modify^14^ to highlight how we feel a general disconnective strategy can be developed more broadly. Such an analysis often helps to highlight useful strategic similarities in apparently different protein chemistries. Thus, for example, whilst other Cβ electrophile tag‐and‐modify reactions have now been explored more broadly in proteins (e.g., Cβ‐Seγ: new nucleophile^15^ or Dha: as a Cβ electrophile for Sγ,^16^ and Seγ^17^) the potential for a Cβ nucleophile to react with a heteroatom electrophile (an inverted polarity disconnection) has not yet been described. In this Minireview, we seek to explore the potential generality of future disconnections (and putative yet unrealized reactions) further. Perhaps strangely, the tools of retrosynthetic analysis have rarely been applied to protein chemistry in a systematic fashion, at least in print. We believe that undertaking such an analysis clarifies the challenges and opportunities inherent in chemical protein modification. By examining a generic polypeptide backbone two broadly defined disconnections are immediately apparent (Scheme 1): amide backbone (disconnections F–H) and side‐chain disconnections (disconnections A–E and I).

### 3.1 Side‐Chain Heteroatom–Carbon Disconnections: Further Developments

In nature, both of these amide backbone and side‐chain disconnections are exploited and accomplished synthetically, essentially solely by carbon–heteroatom bond formation (Scheme 1, disconnections F and I). Hence, both nature and the early work in chemical mutagenesis exploit(ed) useful natural polarities and an oft‐touted^18^ cue to reactivity. This polarity can also provide benefits in chemoselectivity by allowing matched selectivities which are highly valuable in the sea^19^ of competing functional groups that can be found in nature (or even in a single biomolecule). Such an intent to use carbon–heteroatom bond‐forming chemistry drives the extensive use of the natural nucleophilicity of cysteine thiol groups^20^ in many bioconjugations (and indeed in natural PTM). The development of site‐directed mutagenesis thus broadened the generality (site selectivity, regioselectivity) of protein chemical modification by allowing natural residues with particular reactivity, such as cysteine, to be introduced flexibly at predetermined sites. Today, sequential use of genetic mutagenesis to install/position a reactive cysteine tag residue, and then thiol‐nucleophile chemistry (in some form) to modify the cysteine tag is perhaps, next to nonselective lysine conjugation, the most common of all synthetic protein modification procedures. In nature, cysteine is itself subject to a number of PTMs, some of which are amenable to installation by direct reaction of the Sγ nucleophile with a Cδ electrophile (e.g., prenylation^21^), and many which are not (e.g., phosphorylation^22^).

Mimicry of a target side‐chain structure (such as a PTM) has been oft‐employed in protein science when the desired residue is inaccessible by current chemical methods. This approach too, has often exploited heteroatom–carbon bond formation to create thia‐Xxx analogues such as thia‐Lys,^23^ thia‐Arg,^24^ or even thia‐homo‐Glu,^25^ thereby allowing sometimes unique mechanistic insight (into for example, p*K*_a_ or geometry) beyond that offered by the limited palette of classical mutagenesis. In some cases, the trace Cys left from native chemical ligation (NCL; see below) has been advantageously exploited,25b and even the degree of the mimicry has been investigated.^26^ Notably, however, careful control of reaction conditions may prove necessary to prevent unwanted over‐reaction on non‐Cys residues (e.g., His. Met^27^).

Such side‐chain mimicry has not been restricted to the use of Cys as a nucleophile. The same principle of exploiting inherent polarity also underpinned the creation of what might be viewed as a near‐chemical mutagenesis, that is, the conversion of Lys into homoArg using isoureas through reaction of an Nω nucleophile with a Cω electrophile,^28^ an unnatural disconnection which shares close similarity to natural disconnections (e.g., Lys acylation).

Notably, despite retrosynthetic analysis of C−X (and all) bonds allowing, in theory, three different synthons from this type of disconnection [two heterolytic (positive‐negative and vice versa) and one homolytic], to date most approaches have focused on the use of charged synthons with natural polarity.^29^ An important and usefully provocative exception is the use of thiol‐ene (strictly thiyl‐ene) chemistry, which takes advantage of the single‐electron character of a thiyl‐radical for alkylation with somophiles (somophiles or SOMO‐philes are used here as a catch‐all for chemical species with affinity for ′open‐shell′ free‐radical species that possess singularly occupied MOs (SOMOs) such as terminal alkenes. In this system, inherent biases of polarity (electrophilic thiyl) allow regioselectivity (terminal thiylation), but unlike heterolytic disconnections do not dictate the mode of disconnection per se.^30^ This feature has thus allowed disconnection not only to a typically unreactive double‐bond Cδ somophile,^30^ but also access to a rare example of a C‐type disconnection (Scheme 1). The installation of an *N*‐acetyl thialysine mimetic of acetyllysine (using an Sγ radical), which is inaccessible by direct alkylation (using an Sγ nucleophile),^31^ also exploits the altered selectivity of radicals in protein chemistry. A fascinating recent report from the group of Payne provides a tantalizing hint at the potential use of C radicals in carbon–heteroatom bond formation in protein modification.^32^ Generation of an alanyl radical on a peptide substrate by deselenization of synthetically introduced selenocysteine and subsequent trapping with an excess of oxone provides access to Ser through a Cβ radical and an Oγ somophile. This reaction allows an alternative homolytic access to an E‐type disconnection (Scheme 1).

It should be noted that in recent years a number of reactions, often formal cycloadditions that are carbon–heteroatom bond‐forming, have been developed for protein modification, and were largely inspired by the click‐chemistry concept.^18^ A number of different goals have informed the design of such reactions, and often those of “labeling” have dominated many aspects of synthetic protein research in chemical biology. The use of mutually selective, compatible reagent pairs with rapid kinetics for heteroatom bond formation has evolved as a potent solution for this application. However, the limited range of natural structures that can be accessed by such an approach still necessitates the investigation of different disconnections to allow access to structures that might more usefully mimic those needed to recapitulate function.

### 3.2 Amide Bond Disconnections: State‐of‐the‐Art and Prospects

The ribosome, for many the most remarkable and useful biological machine in nature, relies upon the amide bond disconnection F (Scheme 3). At the ribosomal center the condensation reaction of an amino‐acid ester with an amine is enhanced and controlled. The logic of designed processes for synthetic manipulation at the translational level (stop codon suppression, auxotrophic incorporation, etc.) thus relies on the amide bond disconnection approach applied through any associated biosynthetic stringency. Thus the fidelity of the system responsible for error‐free protein synthesis, necessary for cellular functioning, also places grave constraints on such protein engineering because of the evolved stringency of both the ribosome and associated amino‐acid/tRNA synthetases. Whilst cell‐free systems and those with removed or modulated error mechanisms may benefit from greater plasticity, this tension is ever present in direct co‐translational methods for the incorporation of artificial amino acids. Nonetheless, some very useful PTM examples (nitro‐Tyr,^33^ sulfo‐Tyr,^34^ various acylated Lys,^35^ phosphoSer^36^) have been successfully accomplished by exploiting associated plasticities and/or selectivities. At their best these successful systems for incorporation can, of course, benefit from the self‐same translational stringency as a mode of selectivity and quality control, but looser systems which create plasticity may also generate unwanted infidelity.^37^

Moreover, stop‐codon suppression can of course allow the smuggling of a ′stump′ for further ′grafting′ as a tag for chemical modification. Opportunities exist in this regard for synthetic divergence by encoding residues which act as precursors to multiple modifications. For example, *p*‐boronophenylalanine can be chemically transformed, post‐expression, to either phenylalanine or tyrosine.^38^

The biocatalytic manipulation of amides is, of course, not restricted to ribosomal catalysis: proteolytic activity and its reversal can in principle be harnessed to achieve similar ends. Such a series of enzymatic reactions has, in fact, been suggested as a generalized means for the conversion of a single amino‐acid precursor into a number of different side chains.^39^ Thus, mutation from Arg to Lys on the protein soybean trypsin inhibitor through incubation with trypsin (leading to selective cleavage of the Arg64‐Ile65 peptide bond) with subsequent addition of an excess of a free amino acid and use of carboxypeptidase B as a catalyst (or use of chemical activation) allowed the ligation of free Lys to the C‐terminus of the N‐terminal fragment. The use of chemical methods valuably broadened substrate scope to any suitably activated amino acid, albeit with the danger of loss of selectivity.

These and other protease‐catalyzed ligations^40^ as enzymatic mutations can be seen as a forerunner to contemporary chemical protein ligation methods, thus directly altering the protein backbone by a cut‐and‐paste approach. Creative application of such an approach, aided by the rapid maturation of ligation technologies, has significant potential for new approaches in chemical mutagenesis. Native‐chemical ligation (NCL; Scheme 3 b)8c is perhaps the seminal example of the utility of chemical approaches to protein synthesis (and thus the incorporation of modifications). Although our focus is on varied methods, some key features that lie behind the success of the reaction are worth considering as potential guidelines for the design of future reactions that rely on other disconnections. Amongst these, the requirement for two chemical recognition events, the initial capture by a free thiol followed by nucleophilic attack of the N‐terminal amine, lies behind the notable selectivity of the reaction. Thus, dual motifs (if found in suitably productive reaction manifolds) may prove beneficial.

Moreover, the wonderful suitability of this dual motif means that, despite more than thirty years of research, NCL typically relies on this reactivity alone (notwithstanding variations of homologated or altered thiol/selenol etc). Therefore, in practice, the strategy is still largely limited to either a Xaa‐Cys or Xaa‐Ala backbone disconnection although numerous dechalcogenative variations from bespoke thiol/selenol amino acids provide other disconnections in principle.^41^ Thus, the chief obstacle to extending the approach lies also in the reliance on this dual motif, that is, the requirement for an N‐terminal thiol peptide moiety as well as the C‐terminal thioester of a second peptide, which can be challenging to synthesize. While many new thiolated amino‐acid building blocks have been reported for peptide synthesis, few are commercially available and their syntheses tend to be involved (although are ever‐improving). The conversion of cysteine into alanine by desulfurization is currently the only extension to the original method that has been widely applied to protein substrates.^41^ Furthermore, expressed protein ligation (EPL),^42^ one of the most useful applications of NCL for the study of medium to large proteins, remains limited to Xaa‐Cys and Xaa‐Ala disconnections when performed with an expressed C‐terminal fragment. New ligations at non‐Cys sites (e.g., Met^43^), as well as new methods for converting cysteine residues into other natural residues post‐ligation (e.g., akin to prior methods used to create mimics,25b using other disconnections shown in Scheme 1) would thus significantly extend the usefulness of ligation strategies. Also, examples wherein an N‐terminal thiol/selenol auxiliary is installed post‐synthetically, are particularly noteworthy^44^ since they too may prove accessible on recombinant proteins, thus extending access to the C‐terminal partner fragment in the EPL methodology.

### 3.3 Prospects in Chemical Mutagenesis: Overlooked Disconnections

Notably, in seeking to highlight current methods, this Minireview has touched on but a handful of the possible disconnections identified in Scheme 1: essentially disconnections E, F, and I. The others remain largely absent or populated by rare examples. To date, side‐chain modification has also largely been limited to carbon–heteroatom linkages, and driven by the perceived need for fast labeling kinetics in applications such as fluorescent labeling in cells. In these applications, where bolting‐on of a label is paramount, the precise nature of the linkage in the side chain used for modification, and thus the identity of the resultant product, are not necessarily of interest. Such carbon–heteroatom chemistry is thus understandably used because it is rapid and operationally simple. Perhaps an even greater limitation is that currently, only a very few carbon–carbon forming reactions which are potentially protein compatible can be performed in aqueous media. However, the chemical construction of the carbon‐rich native amino‐acid side chains in situ as well as most of their post‐translationally modified variants, in a target‐driven fashion, will necessitate carbon–carbon bond disconnections (such as disconnections A or B (Scheme 1), amongst others).

The C_α_−C_β_ disconnection (A) results in a glycine‐type species as the protein‐associated synthon. At present, no reactions have been reported for the functionalization of glycine in proteins. However, glycine modification is well‐established in the field of peptide chemistry, with reported approaches utilizing glycine cations, anions/enolates, and radicals, including asymmetric variants of the reaction. Unfortunately, few of the reported reactions are sufficiently benign to be readily transferred to a protein context, with most requiring organic solvent, strong base, or elevated temperatures well beyond those either accessible in water or in proteins. Furthermore, as glycine is one of the most abundant amino acids in proteins, opportunities for site‐specific modification are likely to be limited in part by the sheer statistics of design and/or selectivity (a problem that also often plagues Lys modification methods). In this context, functionalization at N‐terminal glycine residues or the use of sequon‐based approaches to position a metal catalyst or activating reagent may provide some requisite chemoselectivity.

The C_β_−C_γ_ disconnection (B) results in consideration of an alanine‐type species. Alanine itself could conceivably be modified by selective C_sp3_−H activation. This activation would likely also require either the identification or design of a polypeptide sequence to facilitate metal coordination and specific C−H activation. Recent work on palladium‐catalyzed transformations, such as Suzuki–Miyaura couplings,^45^ has shown that transition‐metal‐catalyzed reactions can be used for protein modification, and more applications of these powerful metal catalysts can be envisioned, although it is likely that C−C bond formation in the creation and manipulation of non‐aromatic side chains will prove a tougher challenge than those shown so far for C(sp^2^)−C(sp^2^) generation.

Particularly, research into the prospects for C−C bond formation using such an approach should be a high priority and make use of the full gamut of disconnective pathways from new and existing tags as synthetic equivalents. Whilst C_sp3_−C_sp3_ formation would provide useful direct access, approaches such as metathesis could conceivably provide indirect access by C_sp2_−C_sp2_ formation and functional‐group interconversion (i.e. reduction) to exploit the same disconnection. Although efficient cross‐metathesis on proteins has, to date, exploited chalcogen‐mediated relay effects^17^, ^46^ (and hence access to longer side chains), multiply relayed methods could be envisaged, for example, for the C_β_−C_γ_ disconnection (B).

One tantalizing opportunity (amongst many) awaits the discovery of synthetic methods for these C−C disconnections. Building the carbon scaffold that dominates amino‐acid side chains will allow the proper synthesis of not only many natural amino acids but also their modified variants. Interestingly, functional chemical recapitulation of the decoration of proteins with PTMs by writer enzymes can be demonstrated with, for example, thia‐analogues of either methylated Lys^22^, 23d or GlcNAcylated^22^, ^47^ or phosphorylated Ser^48^. However, native (e.g., C−C) linkages would allow true emulation using tag‐and‐modify chemistry, in which a uniquely reactive functional group is introduced into the protein (the synthon tag), which is then modified by selective chemical functionalization to bring in, through the reagent, not only the amino‐acid side chain but also any chosen attached modification. Ready methods for diversely creating many variants at one site from a single tag/synthon protein intermediate can then be envisaged. The synthetic challenges for any method that addresses disconnections A and B (Scheme 1) are significant and exciting in their own right. Thus, any method that allows these disconnections, either A or B, will also face multiple challenges: 1) sufficient reactivity in aqueous/biological milieu; 2) selective bond formation; 3) sufficiently mild reaction conditions under which both protein function and modification function are retained after reaction. Many existing reactions that one can envisage, fail. The solution to such a challenge will likely entail dual development of entirely new reactions with retrofitting of partially forgotten approaches (as highlighted by this Minireview).

## 4 Conclusion

The field of chemical mutagenesis stands poised for a resurgence. The explosion of interest in protein modification chemistry, coupled with the rapid maturation of ligation and carbon–heteroatom bond‐forming approaches, indicates the field is ripe for a new challenge. Natural PTMs are one of the outstanding challenges in protein modification, with the current methods to access these residues in a target‐driven fashion still lacking in key respects. Currently, it does not seem possible to efficiently introduce very small (such as monomethylation of Lys) or overly large (such as GlcNAcylation of Ser) modifications by intervention in the translational machinery. This range leaves enormous opportunity for the discovery of new chemical synthetic methods in the mechanistic dissection of biology. We hope that the somewhat simplistic (but perhaps nonetheless informative) retrosynthetic analyses suggested herein highlight just how many opportunities remain unexplored, and will encourage the investigation of new methods.

The utility of the products of such synthetic methodology seem immediately apparent: not only the creation of a new generation of artificial protein drugs,^49^ but it may not be too much to envisage the development of a vibrant, long‐lived, associated chemical industry, much as current chemistry still bears the strategic hallmarks of the utilization of coal tar. In our own opinion, although discussion of synthetic biology is currently dominated by the exploitation of indirect methods from nucleotides, only when the vision of chemical mutagenesis is achieved and when we can flexibly reprogram the functional molecules of life (proteins, sugars, lipids), can we speak of a true synthetic biology to match the development of synthetic chemistry in the last century.

## Biographical Information

*Thomas Wright obtained his B.Sc(Hons) in chemical and biological sciences from the University of Auckland in 2012 working with Margaret Brimble. For his honors work, he developed the use of thiol‐ene chemistry for the synthesis of lipidated peptides as vaccine candidates. In 2013 he began his D.Phil. with Prof. Benjamin G. Davis at the University of Oxford, where he is currently developing new reactions for the chemical mutagenesis of proteins*.

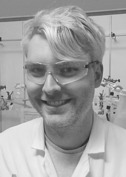

## Biographical Information

*Dr. M. Robert J. Vallée studied chemistry at the Universität Hamburg where he did his Diploma thesis in the group of Prof. Paul Margaretha. He obtained his Ph.D. in 2013 at the Freie Universität Berlin under the guidance of Prof. Christian P. R. Hackenberger. In 2014 he joined the group of Prof. Benjamin G. Davis as a postdoctoral researcher at the University of Oxford, where he worked on different projects, one of which focused on the chemical mutagenesis of proteins*.

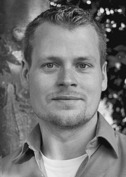

## Biographical Information

*Prof. Benjamin G. Davis received his B.A. (1993) and D.Phil. (1996) at Oxford where he studied carbohydrates with George Fleet. After influential periods at the Universities of Toronto and Durham, he moved to Oxford in 2001 and was promoted to Professor in 2005. His research centers on the chemical understanding and exploitation of biomolecular function with an emphasis on carbohydrates and proteins. He is Editor‐in‐Chief of Current Opinion in Chemical Biology and a Senior Editor for ACS Central Science (2014). In 2015 he was elected to the Royal Society*.